# Intention to work among outpatients with malignant neoplasms, ischemic heart disease, and cerebrovascular disease

**DOI:** 10.20407/fmj.2021-025

**Published:** 2022-05-25

**Authors:** Rumi Seko, Miyuki Kawado, Hiroya Yamada, Shuji Hashimoto

**Affiliations:** 1 Faculty of Nursing, Fujita Health University, School of Health Sciences, Toyoake, Aichi, Japan; 2 Department of Hygiene, Fujita Health University, School of Medicine, Toyoake, Aichi, Japan

**Keywords:** Employment, Malignant neoplasm, Ischemic heart disease, Cerebrovascular disease, Comprehensive Survey of Living Conditions

## Abstract

**Objectives:**

Employment support for working age people with disease is important. We investigated the intention to work among outpatients with malignant neoplasms, ischemic heart disease, and cerebrovascular disease.

**Methods:**

We used anonymous data from the 2007, 2010, and 2013 Comprehensive Survey of Living Conditions in Japan, a self-administered nationwide questionnaire survey. Data for 154,445 participants (76,059 men and 78,386 women) aged 20–64 years were analyzed using logistic regression models adjusted for covariates.

**Results:**

The number of outpatients with malignant neoplasms, ischemic heart disease, and cerebrovascular disease was 851, 1,037, and 716, respectively. The adjusted odds ratio for not working in people with the intention to work was significantly higher among outpatients with the three diseases than among non-outpatients, for both men and women. The adjusted odds ratio for intention to seek permanent work in unemployed people with the intention to work was lower among outpatients with cerebrovascular disease than among non-outpatients for men (p=0.093), and was significantly higher among outpatients with malignant neoplasms than among non-outpatients for women (p=0.007).

**Conclusions:**

This study identified a high proportion of unemployed people with the intention to work among outpatients with these three diseases, and suggests that there are disease-associated differences in employment type sought.

## Introduction

Malignant neoplasms, ischemic heart disease, and cerebrovascular disease are major diseases that have a substantial effect on health and lifestyle.^1^ Because the mortality rate for malignant neoplasms, ischemic heart disease, and cerebrovascular disease is declining, the number of patients who must live with these diseases is increasing, and more attention is being paid to patients’ quality of social and work life.^2^ Employment support is one of the most important measures for working age people with these diseases.^3,4^ In Japan, the need for employment support for patients is described in the Basic Plan to Promote Cancer Control Programs and the National Plan for Promotion of Measures against Cerebrovascular and Cardiovascular Disease.^5,6^

There have been many studies on work and health.^7–12^ It has been reported that people with malignant neoplasms, ischemic heart disease, and cerebrovascular disease have a low employment rate, and that work content is restricted in people with a history of cerebrovascular disease.^7,10–12^ However, few studies have examined the intention to work and the type of employment sought by unemployed people with these diseases.^8,13^ Most people with such diseases are inpatients or outpatients at medical institutions. Because inpatients often cannot work, we investigated the intention to work in outpatients with these three diseases.

## Methods

### Participants

We used anonymous data from the 2007, 2010, and 2013 Comprehensive Survey of Living Conditions in Japan, which is a self-administered questionnaire survey administered to approximately 700,000 people in randomly selected households nationwide each year.^14^ Data from approximately 90,000 people were resampled to anonymize the original data for each year. Of the total sample of 279,267 participants over 3 years, 116,474 people aged 0–19 years and ≥65 years, and 8,348 with no information on work, intention to work, or diseases, were excluded. The final sample comprised 154,445 participants (76,059 men and 78,386 women) aged 20–64 years.

Data were provided with permission from the Japanese Ministry of Health, Labour and Welfare under Article 36 of the Statistical Act. The study protocol was approved by the ethics review committee of Fujita Health University (approval no. HM20-411).

### Diseases

Data on outpatients with diseases were collected using the following two questions: “Do you presently visit a hospital, clinic, or a facility for Japanese traditional massage, acupuncture, moxibustion, or judo-orthopedics for diseases or injuries?” and “What are your diseases or injuries?”^14^ The second question was a multiple-choice question with response options that included 40 diseases or injuries, including malignant neoplasms, ischemic heart disease, and cerebrovascular disease.

### Work, intention to work, and other variables

We used data on work and intention to work. In the surveys conducted in June 2007, 2010, and 2013, participants were asked “Did you work to obtain income in May?”^14^ Participants who responded “Did not work” were asked “Do you intend to work to obtain income?” Those who answered “Yes” were asked about what type of employment they were seeking (permanent work, temporary/contract work, part-time work, and other).

We also collected data on sex, age, and marital status. Age was categorized as 20–29, 30–39, 40–49, 50–59, and 60–64 years. Marital status was categorized as currently married, never married, and divorced/widowed.

### Statistical analysis

We assumed that people who were currently working had an intention to work. The proportion of those who worked and did not work in people with the intention to work was calculated for the following four groups: outpatients with malignant neoplasms, ischemic heart disease, and cerebrovascular disease, and people who answered “No” to the above-mentioned question about whether or not they visited medical institutions (non-outpatients). Non-outpatients did not include inpatients. The proportions of different types of employment sought by unemployed people with the intention to work were calculated for these four groups.

The odds ratios and 95% confidence intervals for unemployed people with the intention to work and for those who sought permanent work in each group of outpatients with malignant neoplasms, ischemic heart disease, and cerebrovascular disease, compared with non-outpatients, were estimated using logistic regression models, with age, marital status, and survey year as independent variables. The level of statistical significance was set at 0.05. All analyses were performed by sex using JMP^®^ 15 (SAS Institute Inc., Cary, NC, USA).

## Results

Table 1 shows the distribution of age and marital status in outpatients with malignant neoplasms, ischemic heart disease, and cerebrovascular disease, and non-outpatients. The number of outpatients with these diseases was 851, 1,037, and 716, respectively. The number of outpatients with the two diseases of malignant neoplasms and ischemic heart disease, malignant neoplasms and cerebrovascular disease, and ischemic heart disease and cerebrovascular disease was 10, 10, and 56, respectively. The proportion of outpatients aged 50–64 years with the three diseases ranged from 65.4% to 86.3%, and was higher than that of non-outpatients (29.3%–29.5%), in both men and women. The proportion of outpatients with the three diseases who were currently married ranged from 71.4% to 83.0%, and was higher than that of non-outpatients, in both men and women.

Table 2 shows the proportions by work and intention to work among outpatients with malignant neoplasms, ischemic heart disease, and cerebrovascular disease, and non-outpatients. Among outpatients with these diseases, the proportion of unemployed people with intention to work was 11.3%–20.7% in men and 23.7%–32.9% in women, and was higher than in non-outpatients. The proportion of unemployed people with no intention to work among outpatients with the three diseases was 11.3%–18.4% in men and 27.7%–42.3% in women, and was higher than in non-outpatients.

Table 3 shows the adjusted odds ratios for unemployed people with intention to work among outpatients with malignant neoplasms, ischemic heart disease, and cerebrovascular disease, compared with non-outpatients. The adjusted odds ratio among outpatients with the three diseases in men and women was 1.79–4.69, and was significantly higher than among non-outpatients.

Table 4 shows the proportion of different types of employment sought by unemployed people with intention to work among outpatients with malignant neoplasms, ischemic heart disease, and cerebrovascular disease, and non-outpatients. Among unemployed people with intention to work, the proportion of those intending to find permanent work was 29.9%–44.4% among male outpatients with the three diseases, and was lower than in non-outpatients. The proportion among female outpatients with malignant neoplasms was 22.0%, and was slightly higher than in non-outpatients. The proportion among female outpatients with the two other diseases was 8.3%–10.8%, and was lower than in non-outpatients.

Table 5 shows the adjusted odds ratios for intention to find permanent work in unemployed people with intention to work among outpatients with malignant neoplasms, ischemic heart disease, and cerebrovascular disease, compared with non-outpatients. The adjusted odds ratio among male outpatients with cerebrovascular disease was 0.62, which was lower than that of non-outpatients (p=0.093). The adjusted odds ratio among female outpatients with malignant neoplasms was 2.15, which was significantly higher than that of non-outpatients (p=0.007).

## Discussion

We found that the proportion of individuals who were unemployed but intended to work among outpatients with malignant neoplasms, ischemic heart disease, and cerebrovascular disease was higher than among non-outpatients, in both men and women. Similar findings have been reported by previous studies.^9–12^ In this study, we confirmed these findings using nationwide surveys in Japan. Some people lose their jobs because of having these diseases; others cannot return to work even if their health improves after treatment.^7,8^ There are many factors associated with preventing turnover and facilitating reinstatement in cases of turnover,^7,8,15^ including factors related to a person’s health status, the work environment, and employment support.^15^ Our results highlight the need for employment support measures for patients with these diseases in Japan.

The adjusted odds ratio for the intention to find permanent work in unemployed people with the intention to work was lower among outpatients with cerebrovascular disease than among non-outpatients in men (p=0.093). Cerebrovascular disease has various sequelae, such as functional limitation, which substantially limits work performance.^7^ Patients with cerebrovascular disease may seek employment types other than permanent work because of their health.

We observed a small difference (i.e., odds ratio close to 1) between outpatients with malignant neoplasms and non-outpatients in the proportion of women intending to find permanent work in unemployed people with intention to work. However, the odds ratio adjusted by age, marital status, and survey year was 2.15, which was significant (p=0.007). The difference between these results may have been caused by the large difference in the proportion of people aged 50–64 years between outpatients with malignant neoplasms and non-outpatients, and the difference in the intention to work between the 20–49 years and 50–64 years age groups. Most of the female outpatients with malignant neoplasms in our study were assumed to be breast cancer patients, as they were aged 20–64 years. Breast cancer is relatively unlikely to cause functional limitations, although it is costly to treat.^8,16^ The relatively large number of outpatients with malignant neoplasms who wanted permanent work in the present study may reflect outpatients’ financial needs, as well as their minor functional limitations.

This study had some limitations. The data were drawn from nationwide surveys conducted in 2007, 2010, and 2013. Each year’s survey data included approximately 90,000 people, but 3 years of survey data were required to obtain sufficient patients for each disease and sex.^14^ The analysis was adjusted for survey year. The data were obtained from self-administered questionnaires. We used commonly asked questions about work and disease in these surveys. However, the responses to these questions may have contained some ambiguity. The surveys did not include questions about disease severity or the site of malignant neoplasms.^14^ The adjusted factors were drawn from previous studies, and included age and marital status.^11,17,18^ Future research should include other factors (e.g., income and education) that may affect intention to work.^17,19^ Possible residual confounding cannot be ruled out in the present study. Additionally, cross-sectional data were used. Disease occurrence and cure, turnover, and reinstatement could not be analyzed. Longitudinal data are needed to analyze these factors.^20,21^

In conclusion, our study identified a high proportion of unemployed people with the intention to work among outpatients with malignant neoplasms, ischemic heart disease, and cerebrovascular disease, and suggests that there are between-disease differences in the type of employment sought.

## Figures and Tables

**Table1:** Distribution of age and marital status in outpatients with malignant neoplasms, ischemic heart disease, and cerebrovascular disease, and non-outpatients

		Outpatients			Non-outpatients
		Malignant neoplasms	Ischemic heart disease	Cerebrovascular disease	
Men	N	265 (100.0)	773 (100.0)	468 (100.0)	54,039 (100.0)
	Age, years				
	20–29	4 (1.5)	3 (0.4)	0 (0.0)	10,908 (20.2)
	30–39	11 (4.2)	19 (2.5)	17 (3.6)	14,509 (26.8)
	40–49	26 (9.8)	84 (10.9)	52 (11.1)	12,808 (23.7)
	50–59	97 (36.6)	307 (39.7)	200 (42.7)	11,110 (20.6)
	60–64	127 (47.9)	360 (46.6)	199 (42.5)	4,704 (8.7)
	Marital status				
	Currently married	220 (83.0)	615 (79.6)	367 (78.4)	33,835 (62.6)
	Never married	23 (8.7)	99 (12.8)	63 (13.5)	18,007 (33.3)
	Divorced/widowed	22 (8.3)	59 (7.6)	38 (8.1)	2,197 (4.1)
Women	N	586 (100.0)	264 (100.0)	248 (100.0)	52,390 (100.0)
	Age, years				
	20–29	10 (1.7)	4 (1.5)	4 (1.6)	10,188 (19.4)
	30–39	44 (7.5)	5 (1.9)	10 (4.0)	14,049 (26.8)
	40–49	149 (25.4)	32 (12.1)	22 (8.9)	12,718 (24.3)
	50–59	259 (44.2)	91 (34.5)	106 (42.7)	10,786 (20.6)
	60–64	124 (21.2)	132 (50.0)	106 (42.7)	4,649 (8.9)
	Marital status				
	Currently married	456 (77.8)	197 (74.6)	177 (71.4)	34,803 (66.4)
	Never married	76 (13.0)	15 (5.7)	13 (5.2)	13,223 (25.2)
	Divorced/widowed	54 (9.2)	52 (19.7)	58 (23.4)	4,364 (8.3)

No (%)

**Table2:** Proportions by work and intention to work among outpatients with malignant neoplasms, ischemic heart disease, and cerebrovascular disease, and non-outpatients

		Outpatients			Non-outpatients
		Malignant neoplasms	Ischemic heart disease	Cerebrovascular disease	
Men					
	People with intention to work				
	Did not work	30 (12.8)	77 (11.3)	79 (20.7)	3,009 (5.7)
	Worked	205 (87.2)	606 (88.7)	303 (79.3)	49,361 (94.3)
	Unemployed people without intention to work	30 [11.3]	90 [11.6]	86 [18.4]	1,669 [3.1]
Women					
	People with intention to work				
	Did not work	99 (23.7)	47 (24.6)	47 (32.9)	8,336 (18.4)
	Worked	319 (76.3)	144 (75.4)	96 (67.1)	36,905 (81.6)
	Unemployed people without intention to work	168 [28.7]	73 [27.7]	105 [42.3]	7,149 [13.6]

No (% of people with intention to work), [% of people without intention to work]

**Table3:** Adjusted odds ratios for unemployed people with the intention to work among outpatients with malignant neoplasms, ischemic heart disease, and cerebrovascular disease, compared with non-outpatients

		Outpatients			Non-outpatients
		Malignant neoplasms	Ischemic heart disease	Cerebrovascular disease	
Men	Adjusted odds ratio for unemployed people with intention to work, (95% CI), p-value	2.40 (1.59, 3.61) <0.001	1.90 (1.46, 2.45) <0.001	4.69 (3.58, 6.16) <0.001	1.00
Women	Adjusted odds ratio for unemployed people with intention to work, (95% CI), p-value	1.79 (1.42, 2.26) <0.001	1.88 (1.34, 2.64) <0.001	2.99 (2.08, 4.29) <0.001	1.00

CI, confidence interval Adjusted variables were age, marital status, and survey year.

**Table4:** Proportions of type of employment sought in unemployed people with the intention to work among outpatients with malignant neoplasms, ischemic heart disease, and cerebrovascular disease, and non-outpatients

		Outpatients			Non-outpatients
		Malignant neoplasms	Ischemic heart disease	Cerebrovascular disease	
Men					
	Type of employment sought in unemployed people with intention to work				
	Permanent work	12 (44.4)	20 (29.9)	26 (39.4)	1,795 (63.0)
	Temporary/contract work	5 (18.5)	30 (44.8)	23 (34.8)	673 (23.6)
	Part-time work	3 (11.1)	5 (7.5)	7 (10.6)	94 (3.3)
	Other	7 (25.9)	12 (17.9)	10 (15.2)	288 (10.1)
Women					
	Type of employment sought in unemployed people with intention to work				
	Permanent work	20 (22.0)	3 (8.3)	4 (10.8)	1,708 (21.4)
	Temporary/contract work	59 (64.8)	27 (75.0)	28 (75.7)	5,730 (71.8)
	Part-time work	1 (1.1)	0 (0.0)	0 (0.0)	175 (2.2)
	Other	11 (12.1)	6 (16.7)	5 (13.5)	363 (4.6)

No (% of unemployed people with intention to work)

**Table5:** Adjusted odds ratios for intention to find permanent work in unemployed people with the intention to work among outpatients with malignant neoplasms, ischemic heart disease, and cerebrovascular disease, compared with non-outpatients

		Outpatients			Non-outpatients
		Malignant neoplasms	Ischemic heart disease	Cerebrovascular disease	
Men	Adjusted odds ratio for intention to find permanent work in unemployed people with intention to work, (95% CI), p-value	1.28 (0.53, 3.06) 0.584	0.69 (0.38, 1.26) 0.230	0.62 (0.35, 1.08) 0.093	1.00
Women	Adjusted odds ratio for intention to find permanent work in unemployed people with intention to work, (95% CI), p-value	2.15 (1.23, 3.74) 0.007	0.77 (0.22, 2.79) 0.695	0.63 (0.20, 1.98) 0.428	1.00

CI, confidence interval Adjusted variables were age, marital status, and survey year.
